# Shocks, stress and everyday health system resilience: experiences from the Kenyan coast

**DOI:** 10.1093/heapol/czaa002

**Published:** 2020-02-26

**Authors:** Nancy Kagwanja, Dennis Waithaka, Jacinta Nzinga, Benjamin Tsofa, Mwanamvua Boga, Hassan Leli, Christine Mataza, Lucy Gilson, Sassy Molyneux, Edwine Barasa

**Affiliations:** c1 KEMRI Wellcome Trust Research Programme, Health Systems and Ethics Research Unit, Bofa Road, Kilifi, Kenya; c2 Centre for Tropical Medicine and Global Health, Nuffield Department of Medicine, University of Oxford, Oxford, UK; c3 Kilifi County Department of Health, P.O BOX 9-80108, Bofa Road, Kilifi, Kenya; c4 School of Public Health and Family Medicine, University of Cape Town, Cape Town, South Africa; c5 Department of Global Health and Development, London School of Hygiene and Tropical Medicine, London, UK

**Keywords:** Health systems, coping strategies, organizational change, decentralization, framework

## Abstract

Health systems are faced with a wide variety of challenges. As complex adaptive systems, they respond differently and sometimes in unexpected ways to these challenges. We set out to examine the challenges experienced by the health system at a sub-national level in Kenya, a country that has recently undergone rapid devolution, using an ‘everyday resilience’ lens. We focussed on chronic stressors, rather than acute shocks in examining the responses and organizational capacities underpinning those responses, with a view to contributing to the understanding of health system resilience. We drew on learning and experiences gained through working with managers using a learning site approach over the years. We also collected in-depth qualitative data through informal observations, reflective meetings and in-depth interviews with middle-level managers (sub-county and hospital) and peripheral facility managers (*n* = 29). We analysed the data using a framework approach. Health managers reported a wide range of health system stressors related to resource scarcity, lack of clarity in roles and political interference, reduced autonomy and human resource management. The health managers adopted absorptive, adaptive and transformative strategies but with mixed effects on system functioning. Everyday resilience seemed to emerge from strategies enacted by managers drawing on a varying combination of organizational capacities depending on the stressor and context.

**Key Messages**

- Middle-level and frontline managers at the sub-national level face a wide range of everyday stressors. These stressors include hardware-related stressors, such as resource and infrastructure challenges, and other software-related challenges, such as low motivation among staff, political interference with managerial responsibilities, unclear roles and reduced autonomy over functions that were previously within managers’ purview prior to devolution.
- Health managers responded to stressors in various ways ranging from buffering stressors (absorptive strategies), making moderate adjustments (adaptive strategies) and making significant changes in structures or processes within the health system (transformative strategies). The effects of these responses varied with some responses having potentially negative consequences, illustrating the challenges of intervening in a complex adaptive system.
- Strategies adopted by health system actors were supported by organizational capacities that include cognitive, behavioural and contextual capacities. These capacities worked in synergy to nurture resilience; hence, one capacity by itself is not sufficient to build resilience.
- Organizational capacities for everyday resilience might be built through health system arrangements that empower health system actors at the sub-national level, reflective practice, providing opportunities for health and non-health actors to connect and leveraging on healthcare worker values in implementing responses to everyday stressors.

## Introduction

Given that health systems face not only shocks but also chronic structural, governance and leadership problems, as well as multiple community and staff demands, Gilson et al. (2017) and Barasa et al. (2017a,b) argue for the exploration of health system resilience using an ‘everyday resilience’ lens (Gilson *et al.*, 2017; Barasa *et al.*, 2017a, 2018). Gilson and colleagues define everyday resilience as ‘the maintenance of positive adjustment under challenging conditions such that the organisation emerges from those conditions strengthened and more resourceful’. They provide some insights on how ‘everyday resilience’ might be nurtured drawing on concepts from vulnerability reduction programmes and organizational theory (Gilson *et al.*, 2017; Lengnick-Hall and Beck, 2005; Lengnick-Hall *et al.*, 2011; Bene *et al.*, 2012).

**Figure 1:**
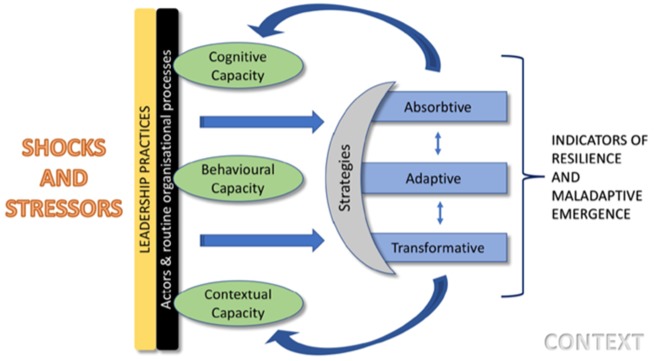
Conceptual framework

Drawing on this previous work (Gilson *et al.*, 2017), we combine concepts of strategies and capacities in our conceptual framework (Figure 1). The framework suggests that strategies adopted in response to a shock or stressor might be absorptive, adaptive or transformative. A chronic disruption of health system functioning is a stressor, while shock refers to an acute disruption. Absorptive strategies buffer the system from shocks and return the system to its state with little or no change in structure; adaptive strategies result in some limited adjustments in the system structure or processes, while transformative strategies result in significant functional or structural changes (Bene *et al.*, 2012). In some cases, strategies employed may result in negative consequences for the health system, termed mal-adaptive emergence (Marion and Bacon, 1999; Gilson *et al.*, 2017).

In response to shocks and stressors, systems draw upon cognitive, behavioural or contextual capacities. Cognitive capacity refers to system ability to interpret uncertainty (sense making) and conceive appropriate solutions including the system’s core values and purpose; behavioural capacity denotes a system’s agency in acting and deploying routine or unconventional responses, drawing on learned resourcefulness and preparedness while contextual capacity is composed of connections and resources that are derived from a combination of deep social capital and broad resource networks (Lengnick-Hall and Beck, 2005). Given the importance of software elements in underpinning an organization’s ‘power to perform’ (Ellocker *et al.*, 2012), we found it useful to characterize the stressors managers faced as either hardware or software related. Aragon distinguishes health system software tangible (skills, knowledge, decision-making processes), software intangible (values, norms, relationships and communication practices) as elements that interact with but are distinct from hardware elements (technology, infrastructure, funding, human resource) (Aragon, 2010; Ellocker *et al.*, 2012).

In this article, we adopt a focus on chronic stressors, because a health system needs to continue to deliver desirable health outcomes in the face of daily stressors (Gilson *et al.*, 2017) and because, potentially, building everyday resilience promotes the system’s ability to respond to acute shocks (Barasa *et al.*, 2017a). We report on the experiences of middle-level (sub-county and hospital) managers and frontline peripheral facility managers in one county, drawing on earlier governance work in that context (Nyikuri *et al.*, 2015, 2017; Tsofa *et al.*, 2017a,b; Barasa *et al.*, 2017b). We investigate the strategies adopted by health managers, and through our analysis, we seek to strengthen the understanding of everyday resilience and the capacities required to build everyday resilience, an important endeavour given the wide recognition of resilience as a legitimate health system objective (Abimbola *et al.*, 2019; Kieny *et al.*, 2017).

Ethical approval for this study was obtained from the authors’ institutes.

### Context: devolution and nationwide health system changes

Previous work in our study setting aimed at understanding governance changes following devolution from a highly centralized national system in Kenya that had eight provinces and 80 districts to a decentralized governance system with 47 semi-autonomous counties (Government of Kenya, 2010; Kramon and Posner, 2011). Following devolution, the national government took up health policy, training and oversight over national tertiary and referral hospitals functions while counties took up management of health service delivery. Owing to political pressure, there was rushed transfer of decentralized processes before counties could establish sufficient capacities to take on new functions, such as financial, commodity and human resources (HR) management (Tsofa *et al.*, 2017a,b; Mccollum *et al.*, 2018). Other changes included a national roll-out of a free maternity services programme aimed at increasing deliveries in health facilities under skilled personnel and a policy directive removing user fees at peripheral facility level aimed at improving access to care for the poor (Nyikuri *et al.*, 2015). The rapid devolution process, arguably an acute shock, coupled with the nationwide policy changes, contributed to significant changes in the health system processes and structures. We illustrate the stressors accompanying these changes and some of the responses by managers, which may demonstrate everyday resilience.

## Methods

### Study design

Our study was conducted under the umbrella of a ‘learning site’ (Gilson *et al.*, 2016; Nyikuri *et al.*, 2015; Tsofa *et al.*, 2017a). A ‘learning site’ is a geographical area in which researchers and health managers have had a long-term relationship and work together to decide on research questions and how these questions might be answered. The researchers are embedded in the health system and have maintained trusting relationships with health system colleagues over time, an approach that enhances the selection of relevant topics and a nuanced understanding of the context researchers’ work in Lehmman and Gilson (2015) and Gilson *et al.* (2016).

### Study setting

Our learning site, Kilifi County, is situated at the Kenyan coast. The population of the county is 1 453 787 as reported in the Kenya Population and Housing Census of 2019 (KNBS, 2019). Table 1 presents other demographic and health indicators for the county.

**Table 1:** Kilifi County demographic and health indicators

Indicator	Kilifi County 2018
Population	
Total	1 498 647
Male	704 089
Female	749 673
Under 5 years	54 518
Under 1 year	259 538
HCWs	
Nurses (per 10 000 people)	4
Doctors (per 10 000 people)	1
Health facilities	
Public	143
Faith based	13
Private	135

Data source: Kilifi CIDP 2018–2022 available at https://www.kilifi.go.ke/library.php and Kenya Population and Housing Census (KNBS, 2019).

### Data collection

Overall, our research activities were primarily qualitative and focused on deepening our understanding of health system stress and resilience. Our data draw on previously published material about early experiences of devolution (Nyikuri *et al.*, 2017; Tsofa *et al.*, 2017a; Cleary *et al.*, 2017) and data collected more recently in our learning site, using participatory approaches that included in-depth interviews, informal observations, informal interviews and reflective practice conducted between September 2017 and November 2018. We also draw on routine health information such as data from the Integrated Human Resource Information System (IHRIS) website and data on staffing gaps extracted from the Kilifi County Integrated Development Plan (CIDP). We also included data from a Service Delivery Indicator (SDI) national report to illustrate commodity, equipment and human resource challenges.

Our learning site approach, involving sustained engagement over a long period of time is considered an embedded form of Health Policy and Systems Research (HPSR) (Olivier *et al.*, 2017; Gilson *et al.*, 2016). Informal observations and interviews are typical in learning sites given the proximity between health managers and researchers and the intention to break down the distinction between researcher and participant (Gilson *et al.*, 2016; Molyneux *et al.*, 2016). Ethical approval for our learning site work included these informal engagements, which serve to build trust and understand the context and nuances of a complex health system (Tsofa *et al.*, 2017a,b; Molyneux *et al.*, 2016). We also conducted reflective practice sessions with health managers and within the research team where we shared our research plans, processes and findings. Reflective practice involves ‘purposeful critical analysis of experiences’ aimed at developing a deeper understanding of a situation (Mann *et al.*, 2009). Reflective practice with managers was guided by a member of the authorship team with experience and training in communication and emotional intelligence for healthcare workers (HCWs). Reflective practice supported reflexivity and was useful for checking resonance of our findings with the health managers (Cleary *et al.*, 2017). Our data include notes from these reflective meetings. We also conducted in-depth interviews with health managers who were purposively selected from different levels of the sub-national health system (county, sub-county, hospital and facility levels). Because health managers are not a homogenous group with just one set of perspectives and priorities, this selection aimed at eliciting a range of perspectives from various health system levels and therefore enhancing the richness of our data and allowing for the emergence of contextual differences in line with our conceptual framework. In-depth interviews were designed specifically to collect data for testing the conceptual framework and to supplement previous information from the longer-term health system governance work under the learning site. The interview guide included questions related to stressors experienced within the health system, how managers responded to these, who they worked with to respond to the stressors and what including who (within/outside the health system) enabled them to respond to these stressors. Because the terms ‘stressors and shocks’ may have different meanings for our study respondents, during in-depth interviews and in reflective meetings, we asked middle-level managers about challenges they experienced within the health system. Interviews were carried out by four members of the authorship team at the convenience of the interviewees, in county and sub-county health managers’ offices and at facility level for hospital and peripheral facility managers. We continued to carry out the interviews until we achieved saturation (Ritchie *et al.*, 2013). We observed two managers’ meetings: one, a monthly facility in-charges meeting facilitated by their sub-county supervisors, and the other, a 2-day planning meeting for county managers who were developing the county strategic plan for 2018–22. We held six researcher reflective meetings. A summary of the data collected is shown in Table 2.

**Table 2:** Data collection summary

Data collection	Number	Details
In-depth interviews	29	In-depth interviews conducted with health managers at various levels of the health system included: county department of health managers (5), sub-county health managers (9), hospital managers (9) and peripheral facility managers (6)
Reflective sessions with managers	3	Notes of reflective sessions with health managers lasting a half to full day
Observations of health managers’ meetings	2	Notes of health managers’ meetings lasting a full day
Researcher reflective meetings	6	Researcher reflective sessions typically lasted 2–3 hr
Routine health information	2	We extracted data on staffing gaps and recruitment strategies from the county human resource website (IHRIS) and the County Integrated Development Plan
National reports with county level data	2	We extracted data on absenteeism, commodity and equipment challenges for Kilifi County from the SDI Report and on population and demographic from the National Census report

### Data analysis

We adopted a framework analysis (Pope *et al.*, 2000) approach driven by our conceptual framework. We immersed ourselves in the data by first reading through the interview transcripts, observation and reflective meeting notes to familiarize ourselves with the data. We applied the conceptual framework to our data by developing empty charts of information that distinguished between different types of interviewees, stressors and responses at different levels of the system. The first and second authors drew on the coded data to fill charts based on the background data, the research objectives, the conceptual framework and new issues emerging from the raw data, combining deductive and inductive approaches. Routine health system data and data from national level reports were useful to enhance our understanding of the different stressors reported by interviewees.

We also held reflective sessions within the research team to share what we learnt in the field and how we should rethink the charts. The initial analysis was discussed within the authorship team leading to the development of a draft paper. The iterative process achieved by many cycles of reflection and triangulation underpin the trustworthiness of our analysis process.

## Results

**Figure 2:**
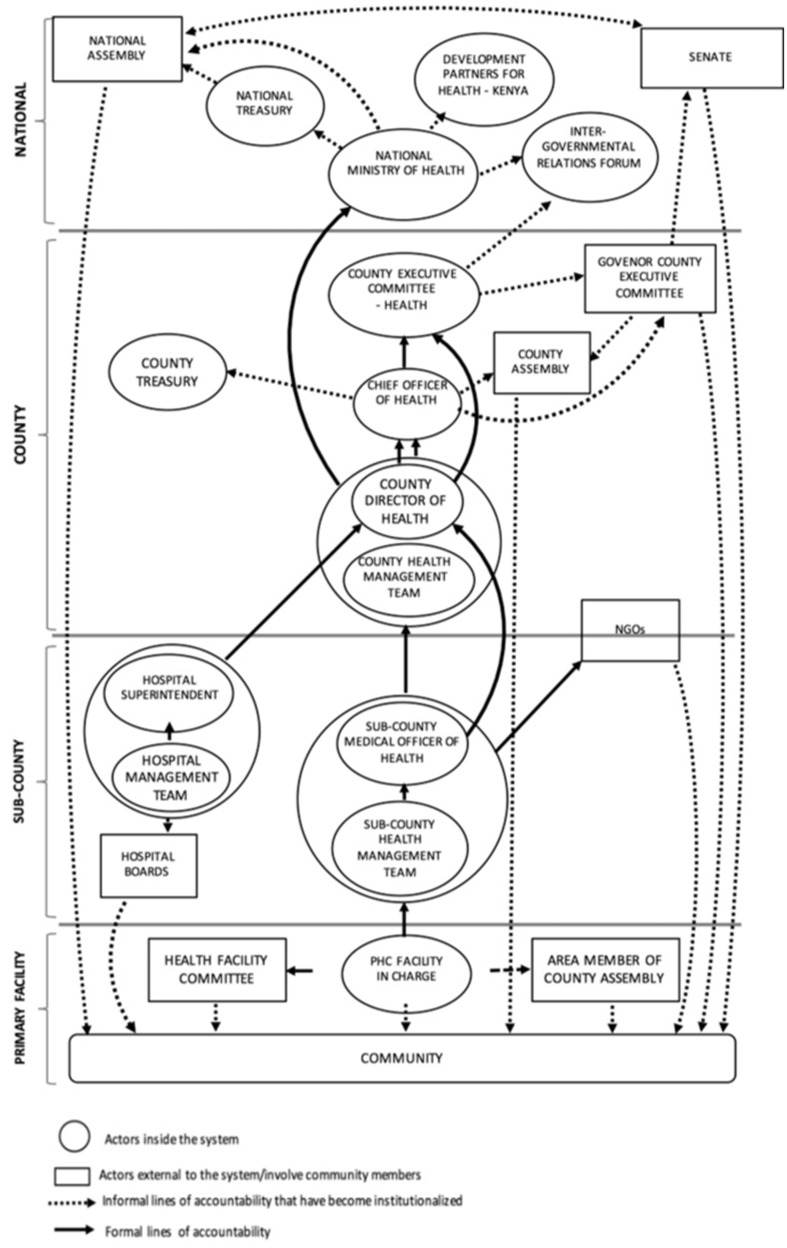
The multiple accountability directions managers in the health system face (adopted from Nxumalo *et al.*, 2018)

In this section, we begin by describing the stressors experienced by health managers at different levels of the health system and, then, we present the strategies in response to these stressors. Woven through our results are descriptions of relationships between actors (Figure 2) and organizational processes. We also present results on effects of actors’ responses on health system goals, given that an action within the health system may build resilience or result in mal-adaptive emergence (Figure 1). Finally, we consider the organizational capacities that enabled these strategies and present this in the discussion section including illustrative examples (Table 7).

### Stressors and shocks

#### Lack of clarity in roles and political interference

At devolution in 2013, a new management level—the County Health Management Team (CHMT) —and new actors—Members of County Assembly (MCAs) and County Executive Committee members—were introduced resulting in a new management structure (Figure 2) and multiple accountability demands for middle-level and peripheral facility managers (Nxumalo *et al.*, 2018; Nyikuri *et al.*, 2015). The Sub-CHMT (SCHMT) struggled to coordinate with the CHMT resulting in the duplication of roles and confusion (Nyikuri *et al.*, 2017). This struggle persisted beyond early devolution experiences; senior county managers frequently communicated directly to facility staff, bypassing the SCHMT or hospital managers. Peripheral facility staff were deployed to facilities, invited to attend training or to answer questions in senior County Department of Health (CDoH) officials’ offices without informing their sub-county supervisors, which undermined the middle-level managers’ authority and affected service delivery due to unstructured absences. One sub-county manager reported:

*We have had a problem of chain of command. Who is answerable to who, even if it’s there …maybe written in circulars it’s not followed, so enforcement of policies has been a problem. There has been a disconnect of how the county and subcounty work, because, sometimes the minister or chief works directly with the subcounty, not through the county team. So, it becomes a problem.*

Some MCAs (locally elected politicians) took an active interest in facility-level processes; they insisted on transfer of staff across facilities, particularly when there had been a complaint from community members. This created anxiety among frontline staff and contributed to mal-distribution of staff. Politicians and senior county government officials also interfered with service delivery by asking hospital and peripheral facility managers to prioritize their patients for care, waive their hospital bills or insisting on referral for patients at peripheral facilities. One peripheral facility manager reported:

*an MCA can force you to refer a patient…because he sees an ambulance here…[a patient] whom you think does not need referral, actually can be managed here. There is a lot of external pressure especially from the political class.*

At hospital level, politicians influenced the employment of support staff leading to unprecedented increases in support staff numbers. Hospitals discontinued pre-existing contracts with cleaning companies to make room for newly employed support staff. The politically connected support staff sometimes declined work allocations, claiming that they had been ‘sent’ to work in specific departments. This created inefficiencies and undermined health managers.

#### Human and financial resource challenges

Commodity shortages and financial flow constraints were among the early experiences of devolution (Tsofa *et al.*, 2017a,b; Nyikuri *et al.*, 2015, 2017). These constraints persisted even after the county government set up structures for commodity and finance management. Peripheral facility managers reported inconsistent disbursement of donor funds; at the time of data collection, funds for two quarters had not been received. At hospital level, control over hospital collections had not been restored to hospitals and biting commodity shortages continued. Facilities operated without a supply of commodities between November 2016 and February 2018 owing to non-payment of the main supplier Kenya Medical Supplies Agency (KEMSA). Table 3 presents findings from the 2018 Kenya Service Delivery Indicator (SDI, 2018) survey that corroborates the view that Kilifi County had inadequate availability of essential medical commodities and equipment. Consequently, staff, citing lack of commodities or equipment, often reported to work late or were absent, leading to delays in patients accessing care, reflecting the emergence of undesirable properties as stressors interacted. A hospital manager reported the consequences of frequent commodity stock-outs:

*You know if there is no water, what happens to a health worker…they’ll not touch a patient because there is no water to wash their hands… the doctor has come to operate, he finds there’s no water, no gloves, no drugs, the patient in the ward doesn’t have the money to buy drugs. Do you think he will come tomorrow? No. He will book clinic day the following week…And maybe next time he won’t be as interested in coming at 8a.m.*

**Table 3:** Health worker, essential commodities and equipment challenges in Kilifi County (SDI, 2018)

Indicator	Kilifi (%)	National average (%)
Health worker absenteeism	60	52.8
Drugs and commodities availability	57	54.1
Essential medical equipment availability	49	50.9

Data source: Service Delivery Indicator, 2018.

Several managers linked staff lateness and absenteeism to the lack of familiarity with the Code of Regulations (COR), a document containing staff norms, usually introduced during staff induction. Managers perceived a lack of support from their supervisors in dealing with staff who had chronic discipline issues. Hospital managers reported that they were the ‘laughing stock of staff’ when no action was taken upon forwarding staff disciplinary problems. They therefore took up staff induction in addition to their other managerial responsibilities. One hospital manager reported:

*…like now there are 31 staff who need induction. So that has affected me because the inductions are not being done, it’s you to do the induction…. So, you leave what you are supposed to be doing to embark on other things [induction of new staff].*

Existing staff shortages (County Government of Kilifi, 2018; Nyikuri *et al.*, 2015)—another hardware-related stressor—was worsened by opening of new departments in hospitals and new peripheral facilities. While a few staff were recruited over time, this was not sufficient to cover existing staffing gaps, as shown in Table 4.

**Table 4:** Staffing gap for medical officers and nurses in Kilifi County

Required doctors^a^ based on population 4.2 per 10 000	629
Actual numbers	99
Percentage of staffing gap	84%
Required nurses^b^ based on population 8.1 per 10 000	1213
Actual numbers	561
Percentage of staffing gap	53.7%

Data source: Kilifi CIDP.

a Medical officers and specialists.

b Bachelor of Science nurses, Kenya Registered Community Health nurses and enrolled community nurses.

Delays in HR processes such as confirmations, promotions, induction and in-service training experienced at devolution (Tsofa *et al.*, 2017a) continued, impacting negatively on staff motivation and retention. In Hospital B, clinical anaesthetists who had not been promoted after completing specialization training left the hospital for better paying counties, worsening understaffing. These HR management issues (tangible software) coupled with resource constraints (hardware) created dissatisfaction among HCWs leading to frequent HCW strikes. In 2017, a 3-month-long doctors’ strike and a 5-month-long nurses’ strike occurred in rapid succession of each other. The strikes crippled service delivery and impacted community members negatively (Irimu *et al.*, 2018; Waithaka *et al.*, 2020). During the strikes, managers extended work hours and some took up clinical roles in efforts to maintain emergency services with skeleton staff across hospitals as described below by a hospital manager.

*At times I would find somebody sleeping outside my door, as a midwife you know what is happening you have to run and do the delivery. At first, I used to get theatre nurses to help, but after some time, they stopped coming. They didn’t want to betray their striking colleagues…It was very difficult…I go home I’m stressed because of what I’ve seen over the day. Then when at home at night the watchman calls you, ‘there’s a mother who is lying here at the gate,’ so you have to go and see how you can help.*

Managers reported managing difficult relationships between striking and non-striking staff, negotiating with senior managers for additional staff while having to make difficult choices about who was prioritized for care. During the strikes, sub-county-level managers already constrained by inadequate resources for support supervision in public facilities extended their support supervision to private facilities, majority of which were offering services beyond their capacity owing to the minimal service delivery in public facilities. Despite concerns about possibly compromised quality of care, the sub-county managers could not ban the facilities from providing care as that would have severely reduced the options available to community members.

#### Reduced autonomy

Managers at hospital and sub-county levels experienced loss of autonomy over planning, resource allocation and hospital collection fees during the early days of devolution in 2013 (Tsofa *et al.*, 2017b; Barasa *et al.*, 2017b). Hospitals could not respond timely to stock-outs due to loss of control over their hospital collections, while the SCHMT continued to grapple with difficulty in visiting facilities for support supervision almost 5 years after devolution. These difficulties included challenges with fuel and broken-down vehicles that went un-repaired for long periods.

Across hospitals and peripheral facilities, managers reported exclusion from decision-making on equipment purchases and project selection, leading to misaligned priorities and low-quality products as reported by one hospital manager:

*Our supervisors run the hospital as if they are micromanaging it… you find the seniors are just constructing a building, you don’t know what this building is and they didn’t ask, what is your priority as a facility? Even procurement of equipment, you find that equipment has been bought, it’s not of quality, but when you say, ‘[You] don’t want it,’ it is still brought*.

### Strategies

In Table 5, we refer to our conceptual framework to unpack the responses to the stressors mentioned above. Some of the responses described in this section were observed in the early days of devolution and continued to be utilized at the time of data collection.

**Table 5:** Strategies adopted in response to chronic stressors (Bene *et al.*, 2012; Gilson *et al.*, 2017)

Stressor	Absorptive (return the system to its previous state with minimal or no effect on its functionality)	Adaptive (the system makes an adjustment to continue functioning)	Transformative (involves significant functional and structural changes within a system)	Effects
Resource scarcity (commodity stock-outs, breakdown or lack of fuel in ambulances)	Borrowing drugs from other facilities and obtaining credit from suppliers. PHC facility managers also had a WhatsApp group where they shared information about drug availability. The hospital pharmacy kept buffer stock that PHC facilities could borrow and, when exhausted, the PHC facilities borrowed from each other Seeking credit from suppliers	Spending at source: This entails spending small amounts of money from hospital collections before banking. This contravenes the law requiring that all collected user fees are banked before spending. Managers keep a record of what the money has been spent on for accounting and audit purposes Re-introduction of user fees in peripheral facilities	Drafting of the FIF bill, which became law in 2016. The bill seeks to restore some autonomy to hospitals by providing a mechanism for the management of hospital funds. New structures were set up, e.g. hospital boards, a county board, collecting accounts for all the hospitals	Borrowing, re-introduction of user fees and spending at source enabled service delivery to continue especially in emergency situations Inherent risks of abuse, legal and reputational consequences for the hospitals (spending at source) Re-introduction of user fees may have reduced access to care for poor patients
Resource scarcity-understaffing	Reorganization of staff shifts and work allocation: Recalling staff from annual leave Staff were transferred from existing departments to other newly opened departments to deal with understaffing. This response was met with resistance by the nursing staff who felt overburdened by having few staff already. The HMT listened to the grievances of the staff but urged them to continue working as the managers made efforts to have more staff employed	Employment of staff on contract basis following requests, meetings and lobbying by hospital managers and representatives of a community organization that was concerned about poor service delivery in Hospital C Task shifting non-technical duties to support staff Extending work hours to include weekends and late evenings Merging departments so that fewer doctors can see more clients across different departments		Reorganization without increasing HCW numbers risked fatigue and burnout among HCWs due to high workload, extended work hours, which could undermine the quality of care offered Employment on contract basis eased workload of existing staff and helped to continue service delivery
HR management challenges—lack of induction that managers perceived to contribute to staff discipline issues, such as lateness, absenteeism	Hospital C HMT invited senior managers from the county level to participate in a staff meeting regarding staff discipline (lateness, absenteeism) and expectations of an employee. This was after the EEC had held frequent meetings with staff from various departments and felt that they needed the support of the CHMT Hospital C HMT made copies of the COR for staff to read. These copies were shared with the various hospital departments for staff to read, to serve as reference material that could guide staff behaviour	The HMT agreed informally that all its members would be expected to correct staff misconduct rather than waiting for the direct cadre manager of the affected staff to handle the issue. This was aimed at ensuring staff behaviour did not affect patient care negatively	Set-up of HR advisory committee to advise on promotion and training needs. The committee meets yearly to identify staff eligible for promotions and then forwards their names to the HR advisory committee at county level. During the 5-month-long nurses’ strike, promotions for staff were fast-tracked and completed in the early weeks of the strike	Getting the support of the CHMT empowered the HMT and reinforced the norms of the organization and was useful for organization strengthening at facility level as it streamlined organizational behaviour by providing boundaries within the COR. The COR could also be used to support facilitative leadership and management Organizational strengthening resulting from shared collective duty by HMT for upholding organization norms Introduction of the HR committee changed processes and helped to break down organizational barriers that slowed down HR processes

EEC, Executive Expenditure Committee; PHC, Primary Health Care.

#### Absorptive strategies

Managers attempted to buffer the strain from some stressors by making minor adjustments in their sub-systems; they borrowed commodities from one another across peripheral facilities and hospitals and, at hospital level, managers obtained supplies on credit. These were practices that managers had adopted pre-devolution and continued with beyond devolution. Due to understaffing, hospital managers transferred staff from existing departments to newly opened departments and recalled staff from leave. One hospital manager reported, ‘we have a talking system’ to describe negotiations with staff to rearrange annual leave to cover staff shortages. One peripheral facility arranged different clinics to run on different days maximizing use of the available space due to infrastructure challenges. These responses as explained by a peripheral facility manager and hospital manager seemed to be motivated by a sense of community with the patients:

*I think it’s think its passion and responsibility, and the love for my clients and my community. I also come from this community, so if this facility doesn’t work it also affects me*.

*These are our people, these are our patients, it’s your mother, it’s my sister, your brother. What do we do, how best can we help them?*

Sometimes, managers’ strategies were resisted by staff. In Hospital B, nurses complained against their manager's effforts to re-distribute them to new departments. This was unsurprising, given that transfer across departments without additional staff increased workload and fatigue and could potentially cause burnout. Hospital managers attempted to balance between the nurses’ concerns and the need to continue service delivery by listening to the nurses’ grievances, with the promise of taking the matter forward to decision-makers, but the managers also appealed to the nurses’ sense of duty to continue working despite the challenges with understaffing.

In response to staff absenteeism, the Hospital Management Team (HMT) in Hospital C held meetings with HCWs to caution them and leveraged the authority of the CHMT by inviting them to speak to HCWs about their conduct and its impact on patient care. At the meeting, hospital managers also shared photocopies of the COR for every staff to read to bridge the gap created by the lack of induction.

In response to political directives to transfer staff, managers attempted to buffer frontline staff by explaining to politicians that unplanned transfers would disrupt service delivery. However, managers were not always successful in protecting their staff from political interference. During a reflective researcher–managers meeting, one hospital manager noted that managers were not ‘powerless’ against political interference:

*While dealing with politicians may be difficult, to communicate and resolve conflict effectively with them, as a manager you should: 1) be fully aware of your roles and responsibilities as the facility (or subcounty) manager, 2) be equipped with the law-knowledge of the constitution and the various acts that affect healthcare/healthcare workers e.g. the Public Finance Act, 3) ensure that you confirm to politicians that your roles and power are anchored in the law. This reveals to the politicians that you are within your rights to make decisions, 4) talk and explain to them about situations and challenges in the facility by lobbying the same politicians 5) ensure you are a good communicator, listen, explain and give direction. Understand the politicians have an agenda.* (Reflective meeting, 16th May 2018).

At sub-county level, managers collaborated with their sub-county administrator to convene a meeting with area MCAs in which they explained to the MCAs how understaffing affected staff availability per shift and how delays in the quarterly supply of drugs contributed to stock-outs. Managers reported a reduction in confrontational visits by MCAs after that meeting.

#### Adaptive strategies

Managers also adjusted processes and ways of working, e.g. peripheral facility managers task-shifted non-technical duties to support staff, freeing HCW time to do clinical duties. A peripheral facility manager explained that, ‘we love to serve’ to illustrate their commitment and team spirit despite staffing challenges. Her staff frequently extended working shifts to ensure the provision of 24-hr services. Facility managers also negotiated with non-governmental organization (NGO) partners to support staffing within their facilities. The NGO-employed staff had specific deliverables for tuberculosis (TB)/human immunodeficiency virus (HIV) care and treatment, but facility managers allocated them elsewhere when TB/HIV clinics were not running, easing the workload of the county-employed staff. The NGO staff also provided some buffer when the health system experienced the 5-month-long nurses strike as they helped to continue prioritized services.

At county level, the County Public Service Board (CPSB) employed nurses on contract, a departure from the usual ‘permanent and pensionable’ terms of employment. Hospital managers reported that they drew on relationships with their supervisors, political actors and communities (Box 1) resulting in nurses being employed on contractual basis in Hospitals B and C. Table 6 shows the proportion of nurses employed on contract to fill staffing gaps across the county between 2013 and 2017 (IHRIS, 2018).

**Table 6:** Terms of employment for nurses in Kilifi County (IHRIS, 2018)

Type of nurse	Definition	Numbers	Percentage
Full-time	Nurses employed on permanent and pensionable terms of service	532	85
Probation	Newly employed full-time nurses awaiting confirmation after probation usually 6 months	61	10
Contract	Nurses employed on contract, varying from 6-month to 3-year contracts	33	5
Total		626	100

Data source: IHRIS available at: http://ihris.or.ke/.

**Table 7:** Organizational capacities underpinning strategies (Lengnick-Hall and Beck, 2005; Lengnick-Hall *et al.*, 2011; Gilson *et al.*, 2017)

Stressor	Response	Cognitive capacity	Behavioural capacity	Contextual capacity
Resource scarcity challenges—frequent and long periods of commodity stock-outs, breakdown or lack of fuel in ambulances	Borrowing drugs and obtaining credit from suppliers. PHC facility managers had a WhatsApp group where they shared information about drug availability. The hospital pharmacy kept buffer stock that PHC facilities could borrow and, when exhausted, the PHC facilities borrowed from each other. (Absorptive)	Hospital and peripheral facility managers borrowed drugs, sought credit to ensure service delivery continued (purpose) and passion for patients (values)	Borrowing drugs and seeking credit are overlearned ‘routine practices that provide a first response’ to commodity stock-outs. Peripheral facility and hospital managers adopted these strategies from the pre-devolution times when they were faced with stock-outs	‘Broad resource networks’ demonstrated by hospitals seeking credit from suppliers whom they maintained relationships with The facility managers of WhatsApp group enabled sharing of information and allowed for exchange of resources
	Spending at source: This entails spending small amounts of money from hospital collections before banking. This is a contravention of the law, which requires that all collected user fees are banked before spending. Managers keep a record of what the money has been spent on for accounting and audit purposes. (Adaptive)	The HMT displayed a ‘strong sense of organizational purpose, “to save life”’ by consistently choosing to buy essential emergency drugs for patients who needed them urgently (when they were out of stock), even though it went against policy	‘Learned resourcefulness’ based on pre-devolution experiences in which staff could spend prior to getting AIE and then reconcile accounts once the AIE was issued. They kept receipts as evidence of spending funds collected before banking	The decision to ‘spend at source’ was informally agreed upon by HMT with the knowledge of the CHMT members (chief officer and county director; county administrator). The decision required risk taking on the part of the HMT but was enabled by the accountability relationships between the CHMT who were aware and the HMT. Different actors therefore drew on the ‘diffuse power and accountability’ relationships within the health system to spend at source
Political interference—politicians frequently visited facilities to confront managers about drug stock-outs and few staff available at the facility contributing to anxieties among staff	The SCHMT, facility managers in collaboration with a sub-county administrator held a meeting with the area politicians to explain issues around staff rota and staff availability per shift and the drug ordering process, including challenges the managers encountered such as delays in receiving commodities. (Absorptive)	Managers’ perception of the stressor (political interference) reflects a change in their mindsets as they reframed the stressor in a problem-solving manner. This enabled them to come up with actions to create awareness among the politicians about facility-level and health system processes that the politicians were not aware of	By inviting politicians to discuss with them the everyday challenges of the facility, the facility and sub-county managers demonstrated ‘counter-intuitive agility’, as they enacted a response differing from normal organizational habits where politicians were often not engaged by HCWs unless they visited the facilities of their own accord	By involving various actors within the health system (the SCMOH, the facility manager) and a representative of the county government (the sub-county administrator), the managers drew on ‘diffuse power and accountability’. The various actors involved in convening the meeting had differing roles and authority, but they all had discretion and the responsibility for ensuring good function of the health system, e.g. the SCMOH has power derived from their managerial role in the health system, while the sub-county administrator is a representative of government administration. This shared responsibility coupled with interdependence enabled the setting up of the meeting to engage MCAs
HR management challenges—lack of induction was believed to contribute to increase in staff misconduct (frequent lateness, absences from the work station)	The Mariakani HMT made copies of the COR. These copies were shared with the various hospital departments for staff to read, to serve as reference material that could guide staff behaviour. (Absorptive)	The managers exhibited a shared mindset based on the ‘values’ of their professions and norms from the code of conduct. Recognizing that actions, such as lateness, absenteeism undermined patient-centred care values they innovatively made copies of the COR book available to everyone at the hospital as material for reference	The HMT’s actions were founded on previous practices of induction (useful habits), where staff were inducted into public sector employment using the ‘code of regulations’. This response allowed for new staff to be introduced to and begin to absorb the culture and values of the hospital	
	The HMT agreed informally that all its members would be expected to correct staff misconduct rather than waiting for the direct cadre manager of the affected staff to handle the issue. This was aimed at ensuring staff behaviour did not affect patient care negatively. (Adaptive)	The HMT drew from a ‘positive conceptual orientation’. They had a strong value system (which was client-centred focus) based on existing norms—the COR and the professional standards of HCWs. This informed their actions to hold meetings and talk to staff to create a sense of collective duty on service provision	The HMT ‘acted counterintuitively’, rather than reinforcing the practice of cadre hierarchy, they departed from the norm by changing usual practice to allow various HMT members to exercise leadership across the organization (hospital)—distributive leadership	By relying on the dispersed influence of various HMT members, each HMT member had the responsibility for ensuring the attainment of an organizational culture where patient needs were put first. This response shared responsibility for actions across the hospital departments, thus drawing on the ‘diffuse power and accountability’ of the various managers in this hospital
Reduced autonomy over hospital user fees	Drafting of the FIF bill, which became law in 2016. The bill sought to restore some autonomy to hospitals by providing a mechanism for the management of hospital funds. New structures were set up, e.g. hospital boards, a county board, collecting accounts for all the hospitals. (Transformative)	Frequent interactions and meetings with different actors helped to achieve ‘shared meaning’. Actors within the health system and outside the health system had different understanding of the purpose of hospital collections. By reporting on the challenges faced by hospital managers due to reduced autonomy over hospital collections, other actors could see the importance of channelling hospital user fees back to hospitals		Drafting of the bill drew on ‘deep social capital’. The interpersonal connections and respectful interactions between the learning site researchers, the CDoH and county government allowed for an environment in which differing views by key actors could be discussed. The learning site researchers (1) highlighted challenges experienced at hospital level due to the centralization of user fee collection at county level with delayed disbursement to hospitals, creating a platform for subsequent discussions and (ii) advised on drafts and in discussion with the County Assembly Respectful interactions eased collaborations as different actors (the CDoH, learning site researchers, County Legal Secretary) worked together to present the bill to the County Assembly and ensure that it was passed
HR management challenges	Set-up of HR advisory committee (Box 2) to advise on promotion and training needs. The HR advisory committee is composed of representatives from clinical and non-clinical departments including CPSB, HR officers, however not every HCW cadre is represented. During the 5-month-long strike period, promotions for staff were fast-tracked and completed in the early weeks of the strike. The committee meets yearly to identify staff eligible for promotions and then forwards their names to the HR advisory committee at county level. (Transformative)	To ensure that the voices of the HCWs were heard, they were included in the HR advisory committee that identified staff for promotions, training, etc. This represents a ‘novel and appropriate’ solution for dealing with the backlog of HR issues, which helped to increase transparency about considerations in budgeting for training, hiring and promotions	The set-up of the HR advisory committee allowed groups that did not previously meet such as CDoH staff, HR and staff from treasury to meet creating a new of managing HR processes that was inclusive and that shifted away from the silo approach where only HR people worked on HR challenges. This represents ‘counter-intuitive action’ away from normal organizing habits. This response enabled greater understanding of the system hiring, promoting transparency in career progression	

AIE, Authority to Incur Expenditure; PHC, Primary Health Care; SCMOH, Subcounty Medical Officer of Health.

##### Mal-adaptive emergence

At devolution, owing to inconsistent disbursement of funds, peripheral facilities reintroduced user fees while hospitals ‘spent at source’, strategies that contravened policy and could have undermined equity and accountability, respectively (Nyikuri *et al.*, 2015, 2017; Gilson *et al.*, 2017; Barasa *et al.*, 2017b). These strategies continued as resource scarcity persisted, despite the potential negative effects. One hospital manager explained:

*So sometimes you are forced, though you know it’s illegal, but sometimes you are forced to spend at source because you can’t leave a patient dying, the vehicle there’s no money for fuel, so you have to spend whatever you have to save life* *.*

Peripheral facility managers felt compelled to go against policy by charging user fees. In consultation with Facility Management Committee members and sub-county supervisors, they reinstated user fees so that they could ‘save life’ and ‘continue service delivery’. Sub-county managers reported that those facilities that did not charge user fees had to send patients away due to a lack of commodities.

#### Transformative strategies

New structures and processes were introduced in response to some chronic stressors. These included the set-up of the HR advisory committee, composed of HCWs’ representatives, officers from HR department and the chief officer from the County Department of Health. The committee brought different actors together, thus breaking organizational barriers to facilitate the resolution of chronic HR management stressors. The committee had mixed results: it achieved improvements in the HR processes, but its effectiveness was reportedly undermined by coordination challenges with the County Treasury, which resulted in payment delays for in-service training. Consequently, managers who had been trained were not certified, compromising their chances of promotion and potentially worsening dissatisfaction among HCWs (Box 2).

The passing of the Facility Improvement Fund (FIF) bill (Barasa *et al.*, 2017b; Tsofa *et al.*, 2017b) was a potentially transformative response to reduced hospital autonomy. The bill was drafted and passed in collaboration with research team members. It aimed at restoring some autonomy to hospitals over their hospital collections. The ‘learning site’ relationship with the CDoH was key in implementing this strategy; research team members shared feedback on hospitals’ reduced autonomy with CDoH senior officials, leading to a series of meetings that brought together departments such as County Treasury, CDoH and the County Legal Secretary. The meetings allowed for shared meaning to be achieved as divergent views on county revenues and hospital collections were discussed. Upon achieving consensus, the bill was drafted, presented to the County Assembly and passed. New structures such as hospital boards and a county board were set up, and hospitals opened collecting accounts where user fees could be deposited for them to access. However, at the time of data collection, funds had not been deposited in the hospital accounts, reflecting the sometimes slow nature of transformations.

## Discussion

This article presents the experiences of health managers in a low-income setting that recently experienced decentralization. We identified stressors spanning across hardware and software health system elements (Aragon, 2010; Sheikh *et al.*, 2011) originating within the health system, such as staff absenteeism and tensions across management levels and others originating outside the health system such as political interference. We also observed interplay of stressors across health system levels, e.g. political interference with staff distribution strained already understaffed health facilities. In addition, we have elaborated a proposed conceptual framework, building on the literature on resilient health systems. Acknowledging the focus on acute shocks in existing health system resilience literature, we instead focused on chronic stressors to demonstrate everyday resilience. In this section, we discuss the underlying organizational capacities that enabled various responses to stressors and consider the utility of our conceptual framework in guiding everyday resilience strengthening.

Our findings suggest complementarity across all types of strategies in addressing chronic stress. For example, in response to resource scarcity, we observed borrowing drugs across facilities—an absorptive strategy—spending at source, an adaptive strategy and passing of the FIF bill, a transformative strategy that led to the creation of new structures and processes (Table 5). This is consistent with other conceptualizations of resilience (Blanchet *et al.*, 2017; Bene *et al.*, 2012) and empirical work testing a capacity-oriented framing of resilience (Alameddine *et al.*, 2019).

To enable these strategies, different underpinning capacities were drawn upon concurrently (Table 7). The links between the capacities varied between stressors, suggesting that the configuration of capacities required for everyday resilience depends on the stressors. Given the complexity of the health system (Begun *et al.*, 2003), the interaction and interdependence across capacities to enable response to stressors is unsurprising. We also observed mal-adapted emergence (Gilson *et al.*, 2017; Marion and Bacon, 1999) in which capacities were drawn upon to enable responses (such as introduction of user fees, spending at source) that might have undermined health system goals in the long run. Such responses did, however, appear to be solving a problem at the time of implementation, reflecting the challenges of intervening in a complex health system and the need to enhance system cognitive capacities to adopt problem-solving approaches that fit the reality of a complex health system.

Our study findings and conceptual framework provide some ideas on how organizational capacities for everyday resilience might be strengthened. Table 7 presents several underlying factors, including elements of organizational capacities (Lengnick-Hall *et al.*, 2011) that influenced deployment of strategies. First, governance arrangements that empower actors to take transformative actions nurture behavioural and contextual capacities. Devolution enhanced contextual capacity by transferring power and accountability for health functions to county level. This also enhanced agency (behavioural capacity) of health system actors to take major transformative actions such as the development of a law to resolve financial autonomy challenges, and the setting up of a body to resolve human resource challenges. This was possible because the Kenyan constitution and accompanying legislation such as the Public Finance Management Act (2012) provides for county governments to take such actions.

Second, creating and facilitating actor networks in health systems, which often operate in silos, appears to nurture contextual capacities. In our study, the learning site (Gilson *et al.*, 2016; Lehmman and Gilson, 2015; Nyikuri *et al.*, 2017) enabled connection of health managers with non-health actors with influence over the health system in the development and passing of the FIF bill (Tsofa, 2018). In response to political interference, facility-level and sub-county managers engaged with local politicians through a forum organized with the support of local public administrators, resulting in a reduction in unplanned confrontational visits from local political actors. The value of interconnectedness in supporting resilience has been described by Blanchet *et al.* (2017) who relate it to a capacity among health system actors to engage with diverse actors. In our study, interconnectedness was useful for obtaining knowledge about factors external to the health system and building an appreciation of the interests, values and perspectives of other actors and leveraging upon these interests for the good of the health system.

Third, creating spaces and opportunities for reflective practice seems to nurture cognitive, contextual and behavioural capacities. Reflective meetings organized by the researchers and managers provided opportunities where managers discussed shared challenges and begun to reframe stressors in a manner that enabled problem-solving allowing managers to practice their power (Gilson, 2016). Reflective sessions provided a safe space for peer support on topics ranging from difficulties with facility-level staff and supervisors to more complex situations such as introduction of new service areas with limited staff. Reflective practice was useful, e.g. in dealing with political interference; managers reported positive changes as they adapted ideas for engagement with political actors that had been suggested by their colleagues. In our study, reflective meetings were supported by the existing learning site. Cleary *et al.* (2017), also adopting an action research approach, described the value of reflective practice in enhancing relationships and building values. As participants in our reflective sessions were mainly sub-county and hospital managers, this might pose a future implementation challenge; reflective practice tends to challenge organizational culture and, so to be useful in achieving organizational transformation, it may require the participation or at least endorsement of decision-makers at a higher level within the health system, to become a legitimate organizational process (Nicolini *et al.*, 2003).

Finally, across the responses enacted, we observed managers drawing on the intangible software of values and communication. Values such as a sense of community with patients and a desire to reduce patient suffering frequently shaped managers’ responses, a finding consistent with the view that a strong value-driven purpose directs the range of choices for action in resilient organizations (Fullan, 2001; Collins and Porras, 1991). These examples are linked to cognitive capacities and demonstrate the value of plugging into system software (Aragon, 2010; Sheikh *et al.*, 2011). Our findings suggest that where managers leveraged staff’s values to enact a response, staff were less resistant to implement responses to stressors.

Overall, our examination of everyday stressors provides some insight into the multifaceted nature of health system challenges and the organizational capacities required to build everyday resilience. We observed interdependence across organizational capacities suggesting that everyday resilience is developed from a mix of cognitive, behavioural and contextual capacities, rather than one capacity being more important than the others. Our research approach may have influenced our findings; by being a platform for reflective practice and facilitating connection of diverse actors, the learning site might have enhanced the contextual capacities of the health system. This is not necessarily a weakness, as it is responsive to recommendations to adopt more participatory approaches for health system research (AHSPR, 2016; Ghaffar *et al.*, 2017).

## Conclusion

Our study demonstrates that an ‘everyday resilience’ lens is applicable to the realities of our health system given the varied chronic stressors in the health system. The conceptual framework used for analysis was useful to demonstrate different types of strategies and the role of organizational capacities in nurturing (for building) everyday resilience. While there is value in describing different types of strategies, we found that consideration of capacities is vital because they underpin the strategies and influence their impacts on resilience or mal-adapted emergence. The findings from this study suggest that nurturing resilience capacities could enhance health system responses to everyday challenges. Actions could include developing reflective practice spaces in health systems, developing and facilitating networks among health system actors, adopting governance arrangements that empower health system actors to take transformative actions and leveraging on HCWs’ values to achieve shared meaning and reduce resistance to strategies that respond to chronic stress.
